# Real-life data in the treatment and follow-up of idiopathic pulmonary fibrosis: A single-center study

**DOI:** 10.5578/tt.20239603

**Published:** 2023-12-04

**Authors:** Aykut ÇİLLİ, Fatih ÜZER, Sena AKANLAR

**Affiliations:** 1 Department of Chest Diseases, Akdeniz University Faculty of Medicine, Antalya, Türkiye

## Abstract

**ABSTRACT**

**
Real-life data in the treatment and follow-up of idiopathic pulmonary fibrosis: A single-center study
**

**Introduction:** *The aim of this study was to evaluate the real-life treatment and follow-up data of patients with idiopathic pulmonary fibrosis (IPF) in a single- center setting.
*

**Materials and Methods:** *The study included consecutive patients diagnosed with IPF who were followed up at the Akdeniz University, between January 1, 2014 and December 31, 2022. Patient information was obtained from the hospital automation system.
*

**Results:** *A total of 227 patients with a mean age of 72.0 ± 8.2 years were included in the study. One hundred sixty-seven patients (73.6%) received pirfenidone while 60 patients (26.4%) received nintedanib treatment. Radiological findings were used to diagnose IPF in 79.3% (n= 180) of cases. Mean duration of antifibrotic treatment was 26.3 ± 19.9 months. Of the patients, 49.8% experienced hospital admissions during the treatment course, with respiratory reasons accounting for a majority of these admissions (33.6%). Disease exacerbation was detected in 26.6% of the patients during the treatment period. At least one side effect was observed in 126 patients (55.5%), with a significant portion of these side effects being mild to moderate (n=79, 34.8%). Disease progression was observed in 21.6% of the patients under antifibrotic treatment. Dose reduction was necessary in 22.9% of the patients, with an average duration of dose reduction of 29 months. Antifibrotic treatment was switched to another medication in 24.2% of the patients. There were no statistically significant differences in baseline forced vital capacity (FVC) levels between the two groups (p= 0.314) while the diffusing capacity of the lungs for carbon monoxide (DLCO) level was higher in the nintedanib group (p= 0.024), and the six-minute walk distance was shorter (p= 0.049).
*

**Conclusion:** *In this study evaluating patients with IPF under follow-up in our hospital, it was observed that the majority of patients consisted of elderly male individuals, frequent hospitalizations were due to respiratory reasons, and both antifibrotic medications were well tolerated with a similar side effect profile.
*

**Key words:** *Pirfenidone; nintedanib; idiopathic pulmonary fibrosis; real-life; disease progression
*

**ÖZ**

**
İdiopatik pulmoner fibrozisin tedavi ve takibinde gerçek yaşam verileri: Tek merkezli bir çalışma
**

**Giriş:** *Bu çalışmanın amacı, IPF’li hastaların gerçek yaşam tedavi ve takip verilerini tek merkezli bir ortamda değerlendirmektir.
*

**Materyal ve Metod:** *Çalışma, 1 Ocak 2014 ile 31 Aralık 2022 tarihleri arasında Akdeniz Üniversitesi Tıp Fakültesi Göğüs Hastalıkları Anabilim Dalında takip edilen, ardışık IPF tanısı konmuş hastaları kapsamaktadır. Hasta bilgileri hastane otomasyon sistemi üzerinden elde edilmiştir.
*

**Bulgular:** *Çalışmaya ortalama yaşları 72,0 ± 8,2 olan toplam 227 hasta dahil edildi ve bunların %76,7’si (174) erkekti. Yüz altmış yedi hasta (%73,6) pirfenidon alırken, 60 hasta (%26,4) nintedanib tedavisi almaktaydı. Olguların %79,3'ü (n= 180) radyolojik bulgula- ra göre IPF tanısı almıştı. Antifibrotik tedavi süresi ortalama olarak 26,3 ± 19,9 aydı. Hastaların %49,8’i tedavi sürecinde hastaneye yatış yaşamıştır ve bu yatışların çoğunluğu solunumsal nedenlere bağlıydı (%33,6). IPF akut alevlenmesi hastaların %26,6’sında görüldü. Yüz yirmi altı hastada (%55,5) en az bir yan etki gözlendi ve bu yan etkilerin önemli bir kısmı hafif ile orta düzeydeydi (n= 79, %34,8). Antifibrotik tedavi alırken 49 hastada (%21,6) hastalık progresyonu gözlendi. Elli iki hastada (%22,9) doz azaltma gerekti ve doz azaltma süresi ortalama olarak 29 aydı. Hastaların %24,2’sinde antifibrotik tedavisi diğer bir antifibrotik ilaçla değişti- rildi. Nintedanib ve pirfenidon kullanan hastalar karşılaştırıldığında iki grup arasında başlangıç zorlu vital kapasite (FVC) seviyelerinde istatistiksel olarak anlamlı farklılık bulunmadı (p= 0,314), ancak akciğerin karbonmonoksit için difüzyon kapasitesi (DLCO) seviyesi nintedanib grubunda daha yüksek (p= 0,024) ve altı dakikalık yürüme mesafesi daha azdı (p= 0,049).
*

**Sonuç:** *Hastanemizde takip edilen IPF’li hastaları değerlendiren bu çalışmada, hastaların çoğunluğunun ileri yaşta erkek bireylerden oluştuğu, sık hastaneye yatışların solunumla ilgili nedenlere bağlı olduğu ve her iki antifibrotik ilacın da benzer yan etki profili ile iyi tolere edildiği gözlemlenmiştir.
*

**Anahtar kelimeler:** *Pirfenidon; nintedanib; idiopatik pulmoner fibrozis; gerçek yaşam verileri; hastalık progresyonu
*

## INTRODUCTION

Idiopathic pulmonary fibrosis (IPF) is a specific form of chronic and progressive interstitial lung disease. It is known to have the worst prognosis among idiopathic interstitial pneumonias, with reported five- year survival rates ranging from 20% to 40% (1). Treatment options include pulmonary rehabilitation, lung transplantation, oxygen therapy, and antifibrotic medications. Oral antifibrotic agents containing nintedanib and pirfenidone provide symptomatic relief and slow down the decline in lung function although they do not provide a complete cure (2).

Pirfenidone is the first approved antifibrotic medication for IPF treatment. It contains phenyl pyridine as its active ingredient and exhibits anti- inflammatory properties. It also acts as an antifibrotic agent by inhibiting the synthesis of cytokines, particularly TGF-β (3). Multinational, randomized, placebo-controlled phase 3 trials, such as ASCEND and CAPACITY, have demonstrated that patients treated with pirfenidone experience less decline in physiological parameters and slower disease progression compared to the placebo group (4,5). Although gastrointestinal and skin-related side effects have been reported with pirfenidone, they are manageable, and the drug’s efficacy in improving survival outweighs safety concerns (6). Nintedanib, which has similar clinical effects to pirfenidone, exerts its action by intracellular tyrosine kinase

inhibition (7). The effectiveness of nintedanib in managing IPF patients has been examined in the randomized, double-blind, placebo-controlled, multinational phase 3 trials known as INPULSIS-1 and INPULSIS-2, as well as in an open-label long- term extension study called INPULSIS-ON (8,9). Like pirfenidone, it also prevents the progression of lung fibrosis and the decline in forced vital capacity (FVC) (8).

Randomized clinical trials may not always represent the real-life patient population accurately. Exclusion of elderly patients and those with specific comorbidities, as well as the selection of patients in milder stages of the disease, in randomized clinical trials, contribute to this difference. With this study, we aimed to share our real-life experiences with patients receiving oral antifibrotic therapy in our clinic.

## MATERIALS and METHODS

All patients diagnosed with IPF and followed at the Department of Chest Diseases, Akdeniz University Faculty of Medicine between January 1, 2014 and December 31, 2022 were included in this study. Patient information was obtained from the hospital automation system. Demographic data, antifibrotic drug use, baseline physiological parameters (FVC, DLCO, six-minute walk test, and minimum oxygen saturation), antifibrotic drug changes, dose reductions, treatment discontinuation rates, radiological findings, hospitalizations during treatment, duration of

antifibrotic use, number of exacerbations during treatment, drug side effects, and severity of the side effects were recorded in the data collection form.

Pulmonary function tests were performed three times, and the best value was considered as the result. Progression was defined as meeting at least two of the following criteria: a decline in FVC of more than 10% from baseline, radiological progression on HRCT, a decline in DLCO of more than 15% from baseline, and worsening symptoms (2). Mild to moderate side effects were considered as those not requiring a change in medication and responding to symptomatic treatment, severe side effects were considered as those requiring a change in medication and not responding to symptomatic treatment, and serious side effects were defined as life-threatening or requiring prolonged hospitalization.

Patients receiving at least one dose of antifibrotic medication were included in the study while those not using any antifibrotic medication, those with incomplete data in their electronic files, and patients diagnosed with progressive pulmonary fibrosis were excluded. IPF acute exacerbation was defined as acute worsening of dyspnea clinically, new bilateral ground-glass opacities and/or consolidation superimposed on a background of usual interstitial pneumonia (UIP) pattern radiologically, in a patient being followed with an IPF diagnosis or newly diagnosed with IPF (10-12). Patients who received antifibrotic treatment for at least one month and

switched to a second antifibrotic drug for any reason were considered as drug changes while the duration of dose reduction represents the average duration of antifibrotic drug use after the dose reduction. The medication was switched in the following situations: in cases of severe side effects, a decline in FVC more than 10% or a 5-10% decline in FVC along with radigoraphic progression. Patients’ files were retrospectively scanned, and disease progression or acute exacerbation diagnoses were made following current guidelines.

The study was approved by the Ethics Committee of the Akdeniz University Faculty of Medicine (Decision no: KAEK-430 Date: 24.05.2023).

## RESULTS

A total of 227 patients were included in the study, with a mean age of 72.0 ± 8.2 years, and 76.7%

(174) of them were males. Figure 1 shows the distribution of 146 patients according to their place of residence in Antalya and its surrounding areas. It can be observed that a significant portion of the patients came from Antalya city center and eastern districts.

Among the patients, 167 (73.6%) received pirfenidone treatment while 60 (26.4%) received nintedanib treatment. Mean duration of the treatment was found to be 26.3 months. The most commonly observed comorbidity was hypertension (n= 101, 44.5%), and

186 patients (81.9%) had at least one additional

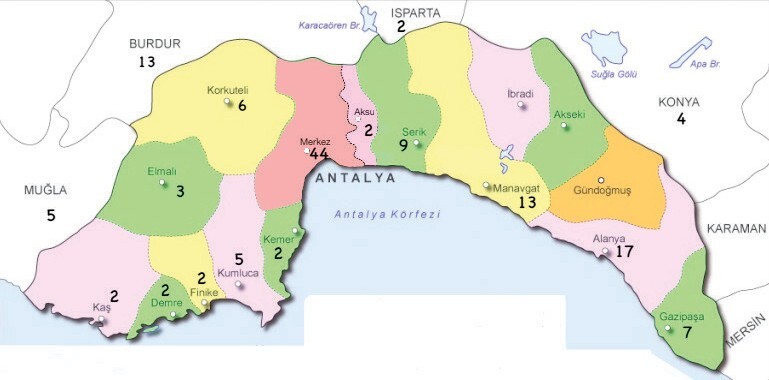

**Figure 1.** The geographic distribution of patients in Antalya and its vicinity.

**Table:** 

**Table 1.** Baseline characteristics of the patients	
**n= 227**	
Age, years (mean ± SD)	72.0 ± 8.2
Male, n (%)	174 (76.7)
BMI, kg/m2	27.145 ± 4.13
GAP (I/II/III), %	35.2/34.8/13.2
Comorbidities, n (%)	186 (81.9)
Hypertension, n (%)	101 (44.5)
CAD, n (%)	75 (33)
DM, n (%)	75 (33)
Others, n (%)	120 (52.9)
Non-smoker, n (%)	66 (29.1)
FVC, L ± SD	2.33 ± 0.77
FVC, % ± SD	69.01 ± 16.66
DLCO, % ± SD	56.7 ± 17.7
6MWT (m), mean ± SD	306.16 ± 120.5
SpO2 (min) ± SS	89.6 ± 6.09
Exacerbations, n (%)	65 (26.6)
Treatment	
Pirfenidone, n (%)	167 (73.6)
Nintedanib, n (%)	60 (26.4)
HRCT findings, n (%)	
UIP	180 (79.3)
Probable UIP	39 (17.2)
Others	8 (3.5)
Surgical lung biopsy, n (%)	47 (20.7)
Switch, n (%)	57 (25.1)
Hospitalization (all causes), n (%)	113 (49.8)
Duration of antifibrotic treatment, months	26.3 ± 19.9
Number of hospitalization, mean	0.99 ± 1.62
Hospitalization (respiratory causes), n (%)	82 (33.6)
Death, n (%)	88 (38.8)
Survey, months (mean ± SD)	52.6 ± 21.2
HRCT: High resolution computerized tomography, UIP: Usual interti- tial pneumonia, BMI: Body mass index, FVC: Forced vital capacity, DLCO: Diffusing capasity of carbon monoxide for lung, 6MWT: Six minute walk test.

disease. Of the patients, 4.4% (10) were receiving anticoagulant therapy, and all of the patients receiving anticoagulant therapy had been initiated on pirfenidone. Of the IPF diagnoses, 78.4% were made radiologically. During the treatment period, approximately half of the patients (49.8%) had hospitalizations, with a significant portion of them

being due to respiratory reasons (33.6%). Exacerbations were detected in 65 patients (26.6%). The mortality rate of patients receiving antifibrotic therapy was 38.8% (88), and the mean survival time was determined to be 52.6 ± 21.2 months. Of the patients, 79.3% (n= 180) received a radiological diagnosis while 20.7% (n= 47) required surgical lung biopsy. The basic characteristics of the patients are presented in Table 1.

Table 2 summarizes the main side effects observed in oral antifibrotic treatment. According to the table, at least one side effect was experienced by 126 patients (55.5%), and a significant portion of these were mild to moderate side effects (n=79, 34.8%). Weight loss (n= 70, 30.8%) was the most commonly observed side effect. Progression was detected in 49 patients (21.6%) under antifibrotic treatment.

Table 3 provides an overview of the dose changes in patients receiving oral antifibrotic treatment. Among the patients, 52 (22.9%) required dose reduction, and the average duration of dose reduction was found to be 1.5 ± 4.6 months. Treatment had to be permanently discontinued in 34 patients (15%). In 24.2% (n= 55) of the patients, the antifibrotic medication was switched to another one. The reasons for the switch were adverse events (40%), decline in FVC >10% (36.4%) and decline in FVC 5-10% and radiographic progression (23.6%). Of the 22 patients who were switched due to SAEs, 14 were switched from pirfenidone to nintedanib (14/167), and eight were switched from nintedanib to pirfenidone (8/60) (p= 0.266). Of the 20 patients who were switched due

**Table:** 

**Table 3.** Dose changes during oral antifibrotic treatment	
**n= 227**	
Drug discontinuation, n (%)	34 (15)
Dose reduction, n (%)	52 (22.9)
Duration of dose reduction, months ± SD	1.5 ± 4.6
Switch, n (%)	55 (24.2)

to decline in FVC >10%, nine were switched from pirfenidone to nintedanib (9/167), and 11 was switched from nintedanib to pirfenidone (11/60) (p= 0.002). Of the 13 patients who were switched due to decline in FVC 5-10% and radiographic progression, nine were switched from pirfenidone to nintedanib (9/167), and four was switched from nintedanib to pirfenidone (4/60) (p= 0.714) (Figure 2).

Table 4 provides a comparison of antifibrotic drugs. The average age of patients using pirfenidone was statistically significantly lower than those using nintedanib (p< 0.001). The nintedanib group had a higher rate of medication switch (p= 0.046), longer duration of dose reduction (p= 0.014), and shorter duration of antifibrotic use (p= 0.029). There was no statistically significant difference in FVC% levels before starting the medication (p= 0.314) between the two groups. However, the DLCO level (p= 0.024) was higher and the six-minute walking distance (p= 0.049) was shorter in the nintedanib group.

## DISCUSSION

In this study, which evaluated patients diagnosed with IPF and followed up at a tertiary care hospital, we found that IPF patients were elderly, with a significant proportion being diagnosed based on radiological findings. It was observed that 26.6% of the patients experienced disease exacerbations during the treatment process, and an equal percentage of patients switched from their current antifibrotic medication to another. Additionally, 55.5% of the patients using antifibrotic therapy experienced treatment-related side effects.

Although the etiology of IPF remains unknown, certain risk factors have been identified. These include advanced age, male sex, and a history of smoking. Both clinical trials and real-world studies in the literature have consistently shown that IPF primarily affects older male individuals with a smoking history (4,5,10,13). However, it has been reported that patients with a positive family history tend to be diagnosed at a younger age (14). Consistent with the existing literature, our study also demonstrated a high proportion of male patients (76.7%) and a significant prevalence of smoking history among the study population (70.9%).

Advanced age and smoking history of the patients diagnosed with IPF increase the likelihood of having comorbidities. These comorbidities can be respiratory

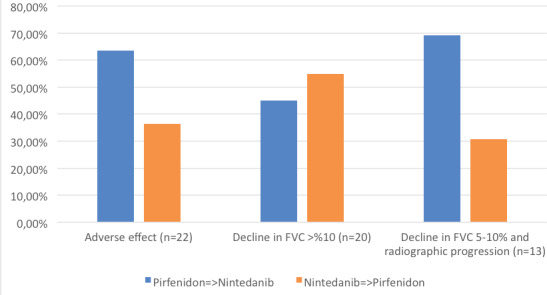

**Figure 2.** Reasons for drug switching.

**Table:** 

**Table 4.** Comparison of antifibrotic drugs			
	**Pirfenidon (n= 167)**	**Nintedanib (n= 60)**	**p**
Age (years)	71.1 ± 8.2	75.93 ± 7.7	**<0.001**
Male, n (%)	140 (83.8)	48 (80)	0.144
Non-smoker, n (%)	45 (26.9)	29 (39.3)	**0.004**
Exacerbation, n (%)	49 (29.3)	16 (26.6)	0.480
HRCT findings, n (%)			
UIP, n (%)	132 (79.0)	49 (81.6)	
Probable UIP, n (%)	29 (17.3)	11 (18.3)	0.961
Others, n (%)	6 (3.5)	3 (5)	
Surgical lung biopsy, n (%)	46 (27.5)	10 (16.6)	0.092
Drug switch, n (%)	32 (19.8)	23 (35.4)	**0.021**
Disease progression, n (%)	34 (20.3)	15 (25)	0.427
Hospitalization(all causes), n (%)	80 (47.9)	33 (55)	0.347
BMI (kg/m2)	26.9 ± 4.2	26.5 ± 3.9	0.710
GAP stage (I/II/III), %	74.2/78.6/60	25.8/21.4/40.0	0.176
FVC ( L)	2.0 ± 0.2	2.3 ± 1.2	0.398
FVC (%)	70.5 ± 14.8	74.3 ± 18.5	0.314
DLCO (%)	55.7 ± 17.5	62.5 ± 17.5	**0.024**
6MWT/m	317.1 ± 114.5	275.6 ± 132.7	**0.049**
Drug discontinuation, n (%)	27 (16.1)	11 (18.3)	0.809
Duration of interruption	0.83 ± 3.22	1.26 ± 4.93	0.452
Dose reduction	43 (25.7)	17 (28.3)	0.921
Duration of dose reduction, months	1.1 ± 3.0	2.8 ± 7.1	**0.014**
Number of comorbidities	1.9 ± 1.4	1.6 ± 1.5	0.139
Number of additional drugs	2.8 ± 2.6	2.6 ± 2.5	0.643
Severity of adverse events			
Mild/moderate	55 (32.9)	24 (40.0)	
Severe	26 (15.6)	14 (23.3)	0.539
Serious	6 (3.6)	1 (1.6)	
Mortality, n (%)	71 (42.5)	22 (36.6)	0.463
Duration of antifibrotic treatment, months	28.9 ± 20.5	22.5 ± 17.7	0.029
HRCT: High resolution computerized tomography, UIP: Usual intertitial pneumonia, BMI: Body mass index, FVC: Forced vital capacity, DLCO: Diffusing capasity of carbonmonoxide for lung.

diseases as well as non-respiratory conditions. The presence of comorbidities can impact the prognosis and treatment adherence of the disease. The most commonly reported comorbidities in the literature among IPF patients include diabetes mellitus, hypertension, obstructive sleep apnea syndrome, pulmonary hypertension, and gastroesophageal reflux. In our study, the most commonly observed comorbidities were hypertension, diabetes mellitus, and coronary artery disease.

The incidence of acute exacerbations in IPF ranges from 5% to 40% per year (10,11,15). The risk of acute exacerbations varies depending on the patient’s ethnicity, age, environmental factors, disease severity, and the definition of acute exacerbation used. The definition of acute exacerbation in IPF has undergone changes over time, particularly after the study by Collard et al. in 2016 (12). The reason for the changes in the definition of acute exacerbation over time is that conditions such as infection, heart failure, and pulmonary embolism can also present with similar

clinical features, and infection cannot be definitively ruled out in every patient. It has been suggested that the Japanese population may be more susceptible to IPF acute exacerbations (16). In our study, the rate of acute exacerbations was found to be 26.2%, which is consistent with other studies in the literature. The presence of specific comorbidities such as gastroesophageal reflux and pulmonary hypertension increases the risk of exacerbations. Additionally, smoking and environmental factors are important factors that contribute to exacerbation risk. The significant proportion of patients in our study with a history of smoking may be associated with an increased risk of exacerbations.

It has been reported that the antifibrotic drugs currently used in the active treatment of IPF are well-tolerated. Studies have indicated that the most common side effects of these drugs are gastrointestinal and cutaneous in nature (17,18). However, these side effects are generally mild to moderate and do not usually require discontinuation or change of medication. In our study, the frequency of any side effects was 55.5%, with the most commonly reported side effects being weight loss, loss of appetite, and diarrhea. The majority of side effects were mild to moderate and responded well to symptomatic treatment. Serious side effects requiring medication discontinuation were observed in only 15% of our patients.

In Türkiye, as in many other countries, pirfenidone and nintedanib are used in the treatment of IPF. Studies have reported that these drugs slow down disease progression, reduce the decline in respiratory function test results, and are well-tolerated in terms of side effects (19,20). In a study by Hanta et al. examining the efficacy and side effect profile of pirfenidone, it has been reported that pirfenidone is well-tolerated and suppresses cough symptoms (19).

Both nintedanib and pirfenidone are effective drugs in the treatment of IPF that slow down disease progression. The selection of which molecule to use in treatment depends on individual characteristics such as the patient’s side effect profile, tolerability, and comorbidities. In our study, it was observed that patients starting nintedanib were older compared to those using pirfenidone. Additionally, the DLCO level was higher and the six-minute walking distance was shorter in the nintedanib group. Moreover, there were no patients receiving anticoagulant treatment in the nintedanib group. In a real-life study comparing

long-term use of pirfenidone and nintedanib conducted by Cameli et al. (21), similar to our study, it has been reported that patients using nintedanib were older, both molecules had similar rates of side effects, and they had similar effects on mortality. The higher average age of the nintedanib group in our study may have contributed to the longer duration of dose reduction in the nintedanib group.

Despite antifibrotic treatment, it has been reported that some patients experience radiological and clinical progression. Due to factors such as incomplete understanding of the etiology of IPF, heterogeneity of the disease, and the inability to eliminate possible environmental causes that may contribute to the disease, progression can occur in some patients despite treatment. In our study, it was found that 21.6% of IPF patients under treatment experienced progression. The lack of a gold standard parameter to be used as a criterion for progression in IPF makes it difficult to determine the true progression rate in patients under treatment. While the progression rate in IPF is around 10% in the first year in phase studies, in real-life studies, it can be much higher, reaching up to 38% in the first year, up to 47% in the second year, and up to 54% after two years (5,8,9,22-25).

In the context of IPF, drug switching primarily occurs due to severe adverse events and disease progression (23,26). In this study, the most frequent reasons for switching were adverse reactions, followed by decline in pulmonary functions. Suzuki et al. have also reported disease progression as the leading cause, with gastrointestinal adverse effects following as the second most common reason (27). Another study has found that both a decline in FVC and intolerable adverse events are equally responsible for the need to switch medications (28).

Data related to survival in IPF is controversial. Some studies report a median survival time of 2-3 years from the time of diagnosis in IPF (1,29). Although phase 3 studies have demonstrated that antifibrotic therapy slows the decline of forced vital capacity (FVC), sufficient effects on overall survival have not been proven (4,5,8,30,31). In one study, it has been reported that patients with GAP stage II and III, under antifibrotic treatment, show a higher survival rate compared to the untreated group (32). In our study, despite the absence of a control group, the average lifespan under antifibrotic treatment was found to be

52.6 months.

The main limitation of our study is its single-center design, which may not represent the general population. Additionally, obtaining data from the hospital automation system may have introduced selection bias.

In conclusion, in this study evaluating IPF patients followed in a university hospital, we found that the majority of patients were elderly males and were frequently hospitalized due to respiratory causes. We observed that the side effect profiles of the two antifibrotic drugs were similar and mild to moderate, and they did not result in discontinuation or dose reduction of the medications. Furthermore, we found that patients using nintedanib were older and had more frequent medication changes.

**Ethical Committee Approval:** This study was approved by Akdeniz University Faculty of Medicine Clinical Research Ethics Committee (Decision no: KAEK-430, Date: 24.05.2023).

## CONFLICT of INTEREST

The authors declare that they have no conflict of interest.

## AUTHORSHIP CONTRIBUTIONS

Concept/Design: FÜ, AÇ, SA Analysis/Interpretation: AÇ, SA, FÜ Data Acqusition: AÇ, SA, FÜ Writing: FÜ, AÇ

Clinical Revision: AÇ, FÜ Final Approval: FÜ

## REFERENCES

1. *
  Ley B, Collard HR, King TE. Clinical course and prediction of survival in idiopathic pulmonary fibrosis. Am J Respir Crit Care Med 2011; 183(4): 431-40. https://doi. org/10.1164/rccm.201006-0894CI.
  *
2. *
  Raghu G, Remy-Jardin M, Richeldi L, Thomson CC, Inoue Y, Johkoh T, et al. Idiopathic pulmonary fibrosis (an update) and progressive pulmonary fibrosis in adults: An official ATS/ERS/JRS/ALAT clinical practice guideline. Am J Respir Crit Care Med 2022; 205(9): e18-47. https://doi. org/10.1164/rccm.202202-0399ST.
  *
3. *
  Schaefer CJ, Ruhrmund DW, Pan L, Seiwert SD, Kossen K. Antifibrotic activities of pirfenidone in animal models. Eur Respir Rev Off J Eur Respir Soc 2011; 20(120): 85-97. https://doi.org/10.1183/09059180.00001111.
  *
4. *
  Noble PW, Albera C, Bradford WZ, Costabel U, Glassberg MK, Kardatzke D, et al. Pirfenidone in patients with idio- pathic pulmonary fibrosis (CAPACITY): Two randomised trials. Lancet Lond Engl 2011; 377(9779): 1760-9. https:// doi.org/10.1016/S0140-6736(11)60405-4.
  *
5. *
  King TE, Bradford WZ, Castro-Bernardini S, Fagan EA, Glaspole I, Glassberg MK, et al. A phase 3 trial of pirfeni- done in patients with idiopathic pulmonary fibrosis. N Engl J Med 2014; 370(22): 2083-92. https://doi. org/10.1056/NEJMoa1402582.
  *
6. *
  Fletcher S, Jones MG, Spinks K, Sgalla G, Marshall BG, Limbrey R, et al. The safety of new drug treatments for idiopathic pulmonary fibrosis. Expert Opin Drug Saf 2016; 15(11): 1483-9. https://doi.org/10.1080/14740338.2016
  *

*.1218470.*

7. *
  Wollin L, Wex E, Pautsch A, Schnapp G, Hostettler KE, Stowasser S, et al. Mode of action of nintedanib in the treatment of idiopathic pulmonary fibrosis. Eur Respir J 2015; 45(5): 1434-45. https://doi. org/10.1183/09031936.00174914.
  *
8. *
  Richeldi L, du Bois RM, Raghu G, Azuma A, Brown KK, Costabel U, et al. Efficacy and safety of nintedanib in idio- pathic pulmonary fibrosis. N Engl J Med 2014; 370(22): 2071-82. https://doi.org/10.1056/NEJMoa1402584.
  *
9. *
  Crestani B, Huggins JT, Kaye M, Costabel U, Glaspole I, Ogura T, et al. Long-term safety and tolerability of nin- tedanib in patients with idiopathic pulmonary fibrosis: Results from the open-label extension study, INPULSIS- ON. Lancet Respir Med 2019; 7(1): 60-8. https://doi. org/10.1016/S2213-2600(18)30339-4.
  *
10. *
  Kishaba T. Acute exacerbation of idiopathic pulmonary fibrosis. Med Kaunas Lith 2019; 55(3): 70. https://doi. org/10.3390/medicina55030070.
  *
11. *
  Collard HR, Moore BB, Flaherty KR, Brown KK, Kaner RJ, King TE, et al. Acute exacerbations of idiopathic pulmo- nary fibrosis. Am J Respir Crit Care Med 2007; 176(7): 636-43. https://doi.org/10.1164/rccm.200703-463PP.
  *
12. *
  Collard HR, Ryerson CJ, Corte TJ, Jenkins G, Kondoh Y, Lederer DJ, et al. Acute exacerbation of idiopathic pulmo- nary fibrosis. An international working group report. Am J Respir Crit Care Med 2016; 194(3): 265-75. https://doi. org/10.1164/rccm.201604-0801CI.
  *
13. *
  Oltmanns U, Kahn N, Palmowski K, Träger A, Wenz H, Heussel CP, et al. Pirfenidone in idiopathic pulmonary fibrosis: Real-life experience from a German tertiary refer- ral center for interstitial lung diseases. Respir Int Rev Thorac Dis 2014; 88(3): 199-207. https://doi. org/10.1159/000363064.
  *
14. *
  Krauss E, Gehrken G, Drakopanagiotakis F, Tello S, Dartsch RC, Maurer O, et al. Clinical characteristics of patients with familial idiopathic pulmonary fibrosis (f-IPF). BMC Pulm Med 2019; 19(1): 130. https://doi. org/10.1186/s12890-019-0895-6.
  *
15. *
  Natsuizaka M, Chiba H, Kuronuma K, Otsuka M, Kudo K, Mori M, et al. Epidemiologic survey of Japanese patients with idiopathic pulmonary fibrosis and investigation of ethnic differences. Am J Respir Crit Care Med 2014; 190(7): 773-9. https://doi.org/10.1164/rccm.201403- 0566OC.
  *
16. *
  Saito S, Lasky JA, Hagiwara K, Kondoh Y. Ethnic differences in idiopathic pulmonary fibrosis: The Japanese perspec- tive. Respir Investig 2018; 56(5): 375-83. https://doi. org/10.1016/j.resinv.2018.06.002.
  *
17. *
  Lancaster L, Albera C, Bradford WZ, Costabel U, du Bois RM, Fagan EA, et al. Safety of pirfenidone in patients with idiopathic pulmonary fibrosis: Integrated analysis of cumulative data from 5 clinical trials. BMJ Open Respir Res 2016; 3(1): e000105. https://doi.org/10.1136/bmjre- sp-2015-000105.
  *
18. *
  Lamb YN. Nintedanib: A review in fibrotic interstitial lung diseases. Drugs 2021; 81(5): 575-86. https://doi. org/10.1007/s40265-021-01487-0.
  *
19. *
  Hanta I, Cilli A, Sevinc C. The effectiveness, safety, and tolerability of pirfenidone in idiopathic pulmonary fibro- sis: A retrospective study. Adv Ther 2019; 36(5): 1126-31. https://doi.org/10.1007/s12325-019-00928-3.
  *
20. *
  Coşkun F. Evaluation of efficacy and safety of pirfenidone
  *

*
200 mg tablets in patients with idiopathic pulmonary fibrosis in a real-life setting. Turk J Med Sci 2021; 51(6): 3082-8. https://doi.org/10.3906/sag-2102-262.
*

21. *
  Cameli P, Refini RM, Bergantini L, d’Alessandro M, Alonzi V, Magnoni C, et al. Long-term follow-up of patients with idiopathic pulmonary fibrosis treated with pirfenidone or nintedanib: A real-life comparison study. Front Mol Biosci 2020;7.
  *
22. *
  Song JW, Ogura T, Inoue Y, Xu Z, Quaresma M, Stowasser S, et al. Long-term treatment with nintedanib in Asian patients with idiopathic pulmonary fibrosis: Results from INPULSIS®-ON. Respirology 2020; 25(4): 410-6. https:// doi.org/10.1111/resp.13647.
  *
23. *
  Cilli A, Uzer F, Sevinç C, Coşkun F, Ursavaş A, Öner Ş, et al. Tolerability and efficacy of second-line antifibrotics in patients with idiopathic pulmonary fibrosis. Pulm Pharmacol Ther 2021; 71: 102099. https://doi. org/10.1016/j.pupt.2021.102099.
  *
24. *
  Tzouvelekis A, Karampitsakos T, Kontou M, Granitsas A, Malliou I, Anagnostopoulos A, et al. Safety and efficacy of nintedanib in idiopathic pulmonary fibrosis: A real-life observational study in Greece. Pulm Pharmacol Ther 2018; 49: 61-6. https://doi.org/10.1016/j. pupt.2018.01.006.
  *
25. *
  Albera C, Costabel U, Fagan EA, Glassberg MK, Gorina E, Lancaster L, et al. Efficacy of pirfenidone in patients with idiopathic pulmonary fibrosis with more preserved lung function. Eur Respir J 2016; 48(3): 843-51. https://doi. org/10.1183/13993003.01966-2015.
  *
26. *
  Vianello A, Salton F, Molena B, Turato C, Graziani ML, Braccioni F, et al. Nintedanib treatment for İdiopathic pulmonary fibrosis patients who have been switched from pirfenidone therapy: A retrospective case series study. J Clin Med 2020; 9(2): E422. https://doi.org/10.3390/ jcm9020422.
  *
27. *
  Suzuki Y, Mori K, Aono Y, Kono M, Hasegawa H, Yokomura K, et al. Switching antifibrotics in patients with idiopathic pulmonary fibrosis: A multi-center retrospective cohort study. BMC Pulm Med 2021; 21(1): 221. https://doi. org/10.1186/s12890-021-01587-3.
  *
28. *
  Ikeda S, Sekine A, Baba T, Katano T, Tabata E, Shintani R, et al. Negative impact of anorexia and weight loss during prior pirfenidone administration on subsequent nintedan- ib treatment in patients with idiopathic pulmonary fibro- sis. BMC Pulm Med 2019; 19(1): 78. https://doi. org/10.1186/s12890-019-0841-7.
  *
29. *
  Raghu G, Collard H, Egan J, Martinez F, Behr J, Brown K, et al. An official ATS/ERS/JRS/ALAT statement: Idiopathic pulmonary fibrosis: Evidence-based guidelines for diagno- sis and management. Am J Respir Crit Care Med 2011;183(6):788-824. https://doi.org/10.1164/ rccm.2009-040GL.
  *
30. *
  Richeldi L, Costabel U, Selman M, Kim DS, Hansell DM, Nicholson AG, et al. Efficacy of a tyrosine kinase İnhibitor in İdiopathic pulmonary fibrosis. N Engl J Med 2011; 365(12): 1079-87. https://doi.org/10.1056/ NEJMoa1103690.
  *
31. *
  Taniguchi H, Ebina M, Kondoh Y, Ogura T, Azuma A, Suga M, et al. Pirfenidone in idiopathic pulmonary fibrosis. Eur Respir J 2010; 35(4): 821-9. https://doi. org/10.1183/09031936.00005209.
  *
32. *
  Platenburg MGJP, van Moorsel CHM, Wiertz IA, van der Vis JJ, Vorselaars ADM, Veltkamp M, et al. Improved sur- vival of IPF patients treated with antifibrotic drugs com- pared with untreated patients. Lung 2023; 201(4): 335- 43. https://doi.org/10.1007/s00408-023-00628-4.
  *

